# MXene potassium titanate nanowire/sulfonated polyether ether ketone (SPEEK) hybrid composite proton exchange membrane for photocatalytic water splitting†

**DOI:** 10.1039/d0ra09935j

**Published:** 2021-03-01

**Authors:** Preeti Waribam, Kanticha Jaiyen, Chanatip Samart, Makoto Ogawa, Guoqing Guan, Suwadee Kongparakul

**Affiliations:** Department of Chemistry, Faculty of Science and Technology, Thammasat University Pathumthani 12120 Thailand ksuwadee@tu.ac.th; Bioenergy and Biochemical Refinery Technology Program, Faculty of Science and Technology, Thammasat University 12120 Thailand; Department of Chemical and Biomolecular Engineering, School of Energy Science and Engineering, Vidyasirimedhi Institute of Science and Technology Rayong 21210 Thailand; Institute of Regional Innovation, Hirosaki University Aomori 030-0813 Japan

## Abstract

A cross-linked sulfonated polyether ether ketone (C-SPEEK) was incorporated with MXene/potassium titanate nanowire (MKT-NW) as a filler and applied as a proton exchange membrane for photocatalytic water splitting. The prepared hybrid composite PEM had proton conductivity of 0.0097 S cm^−1^ at room temperature with an ion exchange capacity of 1.88 meq g^−1^. The hybrid composite proton exchange membrane is a reactive membrane which was able to generate hydrogen gas under UV light irradiation. The efficiency of hydrogen gas production was 0.185066 μmol within 5 h for 12% wt of MKT-NW loading. The results indicated that the MKT-NW/C-SPEEK membrane is a promising candidate for ion exchange with hydrogen gas evolution in photocatalytic water splitting and could be applied as a renewable source of energy to use in various fields of applications.

## Abstract 2

A cross-linked sulfonated polyether ether ketone (C-SPEEK) was incorporated with MXene/potassium titanate nanowire (MKT-NW) as a filler and applied as a proton exchange membrane for photocatalytic water splitting.

## Graphical abstract

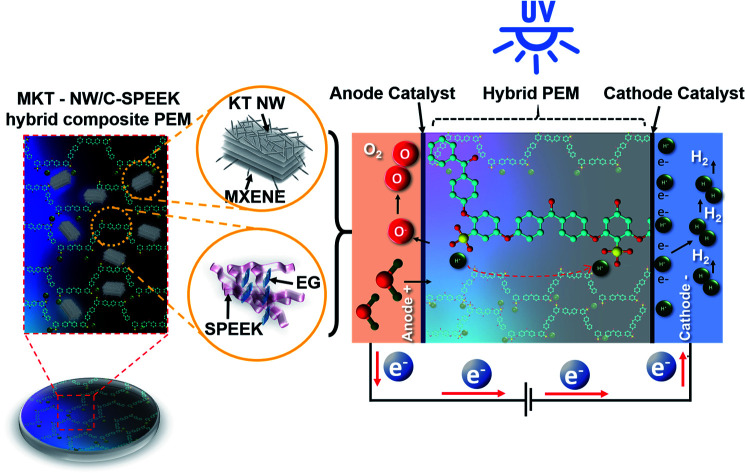

## Introduction

The energy crisis, environmental pollution, climate change, and global warming are some of the crucial environmental problems in the modern world due to the emission of various hazardous byproducts into the environment. The combustion in the automobile engine produces carbon dioxide (CO_2_) and carbon monoxide (CO) gases causing hazardous health problems to humans and, it is one of the major causes of air pollution. Many researchers are working on developing new techniques to produce clean energy which is challenging. Also, providing a realistic solution for the current energy demand is much needed. The use of hydrogen gas (H_2_) as a clean energy source is one of the modern concepts as H_2_ is clean as well as energy-efficient. One of the best ways to produce clean and ecofriendly hydrogen is through a process called photocatalytic water electrolysis or also known as an artificial photosynthesis.^1^ In this process, sunlight is utilized in the presence of photocatalyst to decompose water into H_2_ at the cathode and oxygen (O_2_) at the anode. It was firstly reported by Fujishima and Honda in 1972, however, H_2_ gas generating *via* green processes and high efficiency are still challenging.^2^

Water electrolysis through proton exchange membrane (PEM) is one of those green methods which can produce H_2_ and is utilized in a wide range of applications. Production of H_2_ occurs by the redox reaction that takes place in an electrolyzer cell. PEM water electrolysis is accrued by pumping of water to the anode side where water is splitted into oxygen (O_2_), protons (H^+^) and electrons (e^−^). These protons transport *via* a proton conducting membrane to the cathode side. The PEM allows only the proton (H^+^) produced at the anode to transfer towards the cathode to produce H_2_. Nafion, a conventional commercialized PEM, had been used extensively for the PEM water electrolysis, which is expensive with complicated synthesis steps and toxic to the environment due to the presence of fluorine. Considering the cost-effectiveness, and mechanical and chemical properties, polyether ether ketone (PEEK) is an alternative hydrocarbon-based polymer that can be used for the PEM water electrolysis by the sulfonation of polyether ether ketone. Cross-linking of the sulfonated polyether ether ketone (SPEEK) membrane using ethylene glycol (EG) as a crosslinker increased chemical and mechanical stabilities and decreased swelling ratio which made it suitable for the application as PEM.^3^ The proton conductivity of SPEEK would be reduced when diols are used as a cross-linker, therefore many researchers have incorporated hygroscopic inorganic metal oxide such as SiO_2_, TiO_2_, Al_2_O_3_ and ZrO_2_,^4^ as fillers to improve physiochemical properties and conductivity of the membrane.

TiO_2_ has useful properties among the above-mentioned metal oxides that can be used as a filler material. It is capable of becoming super hydrophilic when exposed to UV light which arises due to the increasing number of hydroxyl groups (–OH) of the TiO_2_ surfaces during UV light irradiation.^5^ TiO_2_ is a nontoxic, cost-effective, abundant material and possesses high chemical stability against the oxidative environment in photocatalytic water splitting.^6^ TiO_2_ particles can be converted to nanowire *via* a hydrothermal reaction using a strong basic solution such as NaOH or KOH solution.^7^ Potassium titanate nanowire is one of the fascinating forms of TiO_2_ that could be utilized as a reinforcing agent as it possesses good mechanical, chemical and thermal stability. Additionally, it was also used as a photocatalyst in water electrolysis.^8^ TiO_2_/graphene nanocomposites have been studied as a filler in PEMs to enhance numerous properties of the membrane.^9^

MXenes are a new versatile inorganic material of 2D transition metal carbides or nitrides, which were produced by selective chemical etching of MAX phases (M is an early transition metal, A is an A-group (mostly IIIA and IVA element) and X is carbon and/or nitrogen). Mostly Ti_3_C_2_T_*x*_ layers are uniformly terminated by –OH and –O– and –F groups after the exfoliation, where T and *x* represent the terminating group and their numbers, respectively.^10^ These surface termination of MXene make them a prominent filler to increase the proton conductivity and physicochemical properties of SPEEK.^11^ MXene has been utilized as an electrocatalyst for H_2_ evolution reaction (HER) in water splitting.^12^ When MXene was employed as a co-catalyst with TiO_2_ in photocatalysis, higher H_2_ evolution has been observed without the addition of any noble metal co-catalyst.^13^ Besides, the addition of co-catalyst with TiO_2_ such as copper (Cu) could obtain a highly active H_2_ production catalyst, Cu/TiO_2_/MXene photocatalyst in which MXene acted as a hole mediator and Cu acted as an electron mediator and reduction co-catalyst. The coupled photocatalyst promoted H_2_ evolution *via* dual-carrier-separation mechanism.^14^

Thus, the present work focuses on the preparation of hybrid PEM, by utilizing sulfonated poly(ether ether ketone) (SPEEK) as a polymer matrix for the PEM water electrolysis. SPEEK with a high degree of sulfonation (DS) had been prepared *via* sulfonation and cross-linked with ethylene glycol (EG) to improve its dimensional stability. To further increase its proton conductivity, inorganic metal oxide; MXene-potassium titanate nanowire (MKT-NW) was embedded into the polymer matrix to prepare MKT-NW/C-SPEEK hybrid PEM. The PEM was prepared by a simple solution casting technique using ethanol and water (1 : 1) as a green solvent. The properties of hybrid composite PEM including photocatalytic activity under UV radiation were characterized.

## Experimental

### Materials and chemicals

Polyether ether ketone (PEEK) powder (particle size ∼ 80 μm) and lithium fluoride (LiF) were purchased from Sigma Aldrich. Absolute ethanol (99.9%), concentrated sulfuric acid (H_2_SO_4_, 98%), potassium hydroxide (KOH) pellets and concentrated hydrochloric acid (HCl, 37%) were obtained from QRëC. Sodium hydroxide (NaOH) pellets and sodium chloride were bought from Merck. Titanium aluminum carbide (Ti_3_AlC_2_ or MAX phase) was supplied from Luoyang Tongrun Info Technology Co., Ltd., China, and Nafion-212 was bought from Wuhan Gaoshi Ruilian Technology Co., Ltd., China. Titanium dioxide (anatase, particle size ∼ 120 nm) was purchased from Ajax Fine Chemicals. All the chemicals were used without purification. Deionized (DI) water (conductivity = 0.067 μS cm^−1^) was used throughout this work.

### Preparation of hybrid composite membranes and characterizations

A hybrid composite membrane consisted of sulfonated PEEK (SPEEK) as a polymer matrix, ethylene glycol as a crosslinker with different amount of MXene–potassium titanate nanowire (MKT-NW) as an active filler. SPEEK was prepared *via* sulfonation of PEEK with conc. H_2_SO_4_ at 50 °C following the literature to obtain orange solid particle of SPEEK.^15^ The degree of sulfonation was characterized by ^1^H-NMR (Bruker AVANCE III HD 600 MHz NMR spectrometer).

For MKT-NW composite, an exfoliated MXene was firstly synthesized *via* an *in situ* production of hydrofluoric acid (HF) protocol using LiF and 37% HCl as an etching agent where the exfoliation was carried out at 50 °C for 48 h.^16^ Potassium titanate nanowire (KT-NW) was prepared by a hydrothermal treatment of TiO_2_ anatase with 10 M KOH solution in a Teflon-lined stainless autoclave at 200 °C for 24 h as reported previously.^17^ MXene potassium titanate nanowire (MKT-NW) was obtained by the hydrothermal reaction of MXene and KT-NW (weight ratio 1 : 1) in DI water/ethanol mixture (3 : 1) using a Teflon-lined autoclave at 120 °C for 3 h. The structure of MXene and MKT-NW was characterized by wide-angle X-ray diffractometry (XRD, Bruker, D8 ADVANCE) with Cu-Kα radiation (*λ* = 1.54 Å) in the range from 5° to 80 °C at the rate of 0.02°/0.5 s.

X-ray photoelectron spectroscopy (XPS) was recorded on JEOL JPS-9010MC with a Mg Kα source (1253.6 eV) at 12 kV and 25 mA. All XPS spectra were measured under a high vacuum pressure of 10–8 Pa at a room temperature. Software for running experiment is SpecSurf ver.1.9.3. The sample was fixed on carbon tape. The analyzed area was a cycle spot with a diameter of 6 mm. The survey scan spectra were measured with a pass energy of 50 eV, a binding energy range of 0–1100 eV and an electron-volt step of 1 eV. The narrow scan spectra were measured with a pass energy of 10 eV and an electron-volt step of 0.1 eV. The obtained spectra were evaluated using JEOL software to obtain the chemical state of the probing elements and elemental composition. All the binding energy values were referenced to the carbon peak C 1s at 284.70 eV.

**Scheme 1:**
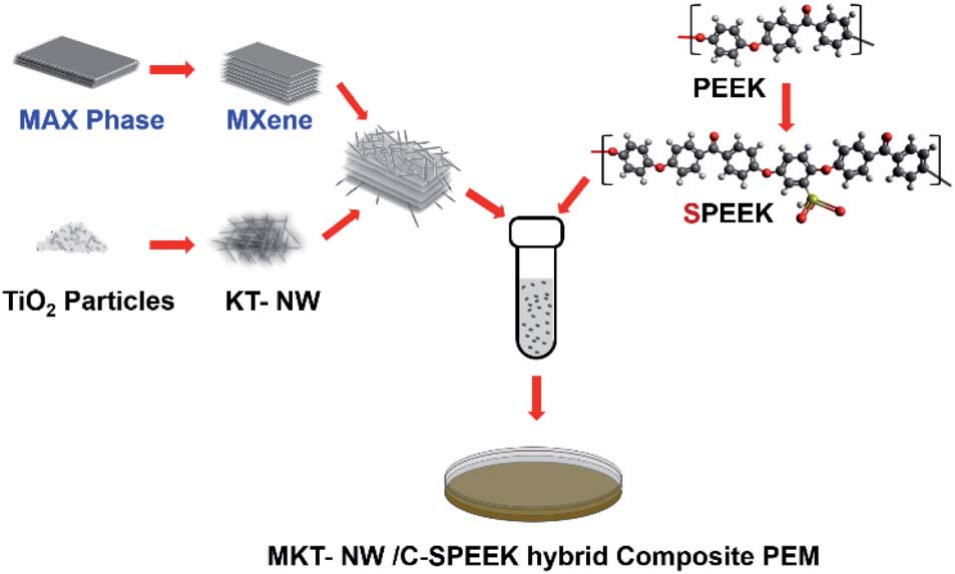
Overall steps involved in the preparation of the composite PEM.

In a typical procedure of the composite membrane preparation, SPEEK particles (5 wt%) were dissolved in ethanol/DI water mixture (1 : 1), then the amount of ethylene glycol was added as a cross-linker and stirred vigorously for 2 h. The MKT-NW suspension (MKT-NW in ethanol/water mixture) was added to the SPEEK solution, then the final mixture was sonicated and stirred vigorously for 24 h, to make the uniform dispersion. The solution was cast on a Petri dish, dried at room temperature, and cured at 120 °C for 24 h to obtain a cross-linking membrane. After that, it was cooled down to room temperature and the membrane was peeled off using DI water and protonated by the immersion in 1 M H_2_SO_4_ for 24 h. Then, the membrane was washed with DI water until neutral and dried at 60 °C for 12 h. The composite membranes were named as MKT-NW_*x*_/C-SPEEK where *x* is the amount of MKT-NW (5 to 20 wt%). Pristine SPEEK membrane, as well as cross-linked SPEEK (C-SPEEK) membrane, were also prepared and used. The overall steps involved in the preparation of the composite PEM is described in Scheme 1.

The chemical structure of the membrane was characterized by attenuated total reflectance-Fourier transform infrared (ATR-FTIR) spectroscopy (PerkinElmer Spectrum 100 FT-IR spectrometer) in the wavenumber range of 4000–500 cm^−1^. The morphologies of the synthesized inorganic powders and composite membranes were characterized by a field emission scanning electron microscope (FE-SEM, model Versa) with energy dispersive X-ray spectroscopy (EDS) mapping. Transmission electron microscope (TEM, JEOL, JEM-2100Plus) was carried out to study the structural change of pristine MXene and after deposition with KT-NW. The elemental distribution in the MKT-NW/C-SPEEK composite membrane was carried out using X-ray fluorescence microscopy (XRF, Horiba, XGT-7200). Dynamic mechanical analysis (DMA) of the membranes were carried out using Mettler Toledo DMA1 analyzer in the tension mode at an oscillation frequency of 1 Hz and the temperature range of 30–260 °C with the heating rate of 3 °C min^−1^. The storage modulus (*E*′), loss modulus (*E*′′) and loss tangent (tan *δ*) were determined.

For the membrane properties, water uptake and swelling area of the membrane were obtained by immersing the membrane in DI water for 24 h, wiped of the excess of water, then weighed and measured the dimension to find the water uptake percentage and swelling percentage by a gravitational calculation. The ion exchange capacity (IEC) value of the membrane was determined using acid–base titration. The sample was immersed in 2.0 M NaCl solution at room temperature for 24 h to complete liberate H^+^ by exchanging with Na^+^. Then, the liberated H^+^ was then titrated against 0.01 M NaOH solution using phenolphthalein as an indicator. The membrane resistance was determined by using an electrochemical impedance spectroscopy (EIS, Corrtest CS310). In this method, the membrane was immersed in DI water for 24 h to make a completely hydrated membrane. The proton conductivity was calculated based on the area, thickness, and resistance of the membrane from EIS measurement.

All calculations and measurements such as the degree of sulfonation, water uptake percentage, swelling percentage, ion exchange capacity (IEC, meq g^−1^), and proton conductivity (*σ*, S cm^−1^) were explained in ESI.†

### Photocatalytic of water splitting testing

**Scheme 2:**
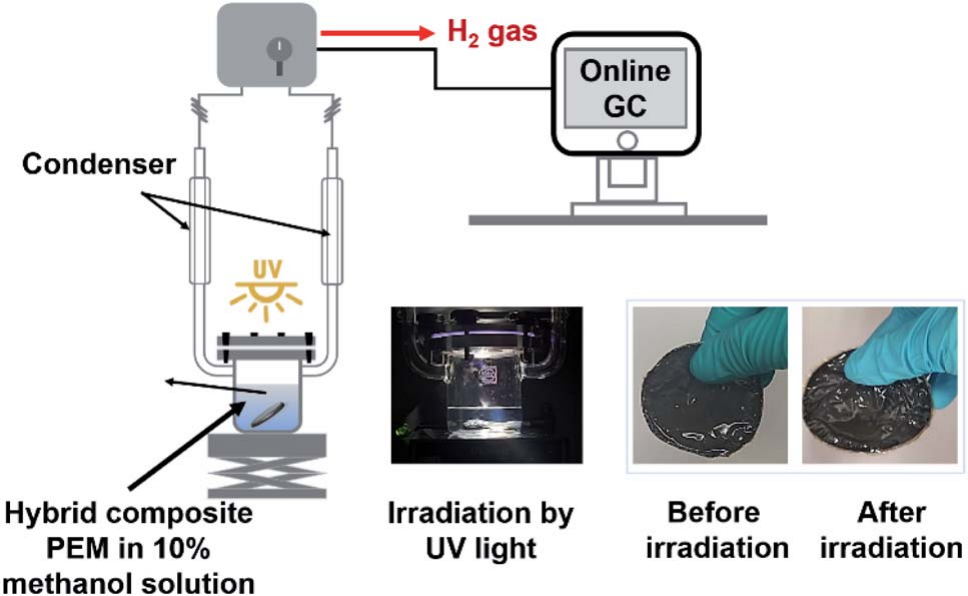
Workstation for photocatalytic activity testing. Photographs of glassware and membranes taken with digital camera.

Photocatalytic activity of a hybrid composite membrane was carried out in a photoreactor by using high-pressure mercury lamp as UV light source (400 W at 365 nm). The amount of H_2_ production was measured by an online gas chromatography (Shimadzu, GC-2010 PlusA BID) equipped with a TCD detector and a 5A molecular sieve packed column using argon as carrier gas. Hybrid composite PEM was immersed in a 10% v/v aqueous methanol solution (120 ml) as a sacrificial reagent for hydrogen production. Before starting the experiment, the sacrificial reagent in the reaction system was purged with argon in the dark environment to remove oxygen completely. UV lamp was set closer to the membrane by exposing only 30% of it with the intensity of 27.1545 W cm^−2^. Later, the light source was moved further from the membrane to expose 100% of it and the light intensity was recorded as 0.0375 W cm^−2^. The workstation set up for testing the photocatalytic activity of MKT-NW/C-SPEEK at room temperature is depicted in Scheme 2.

## Results and discussion

### Structural and material characteristics

**Fig. 1:**
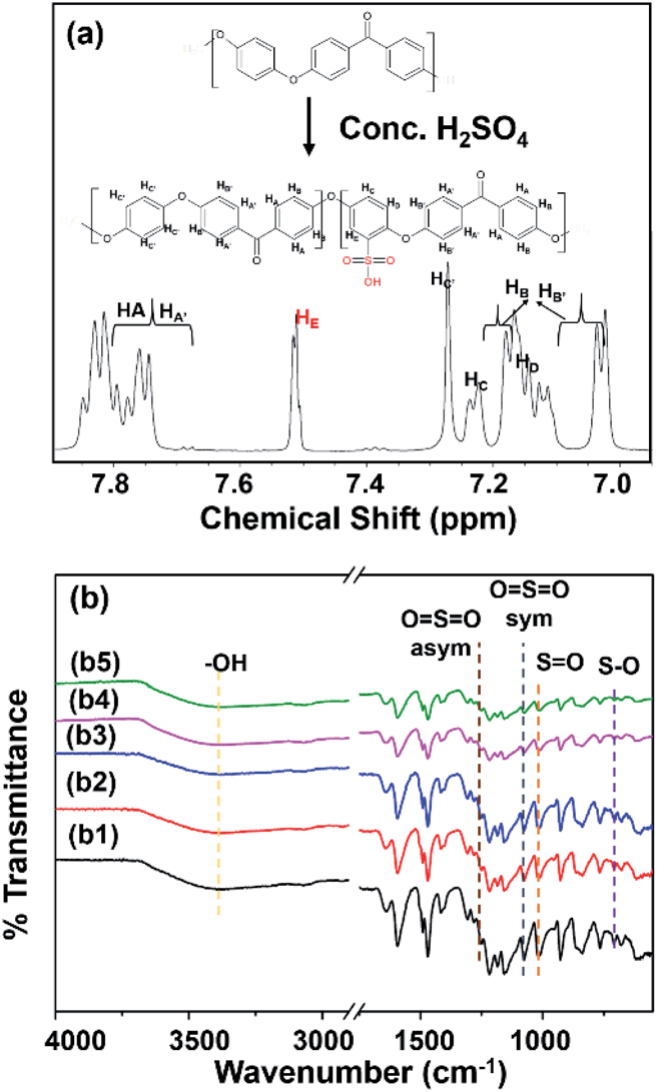
(a) ^1^H NMR spectrum of SPEEK particle (in 25 °C using DMSO-d_6_) and (b) ATR-FTIR spectra of (b1) SPEEK, (b2) C-SPEEK, (b3–b5) 5%, 10%, and 12% MKT-NW/C-SPEEK hybrid composite PEM.

SPEEK formed by the electrophilic substitution reaction of PEEK with concentrated sulfuric acid.^3,18^ From Fig. 1(a), the degree of the sulfonation of SPEEK was 73.10%. FTIR spectra showed characteristic peaks of sulfonyl group (–SO_3_H) at 1250 cm^−1^ (O=S=O asym), 1075 cm^−1^ (O=S=O sym), 1010 cm^−1^ (S=O), and 707 cm^−1^ (S–O), which were introduced into the hydroquinone segment of the PEEK polymer chain (Fig. S1†).

From the FTIR spectra of all prepared MKT-NW/C-SPEEK composite membranes as depicted in Fig. 1(b), the hydroxyl group (–OH) at 3394 cm^−1^ had been shifted to lower wavenumber for C-SPEEK sample due to cross-linking between –SO_3_H of SPEEK and –OH of ethylene glycol, the crosslinker.^19^ Besides, the characteristic peak of –SO_3_H remained unchanged and no new peak observed which implied that there was no chemical reaction and only interfacial interaction between C-SPEEK and MKT-NW.^11^ However, the decrease in the peak intensity of –SO_3_H was observed which may be due to physical interaction of MKT-NW composite with –SO_3_H of SPEEK. For the incorporation of MKT-NW in C-SPEEK, the –OH functional group intensity slightly decreased which may be attributed to the extensive hydrogen bonding interaction between –OH terminated MKT-NW surface and –SO_3_H of C-SPEEK.^20^

**Fig. 2:**
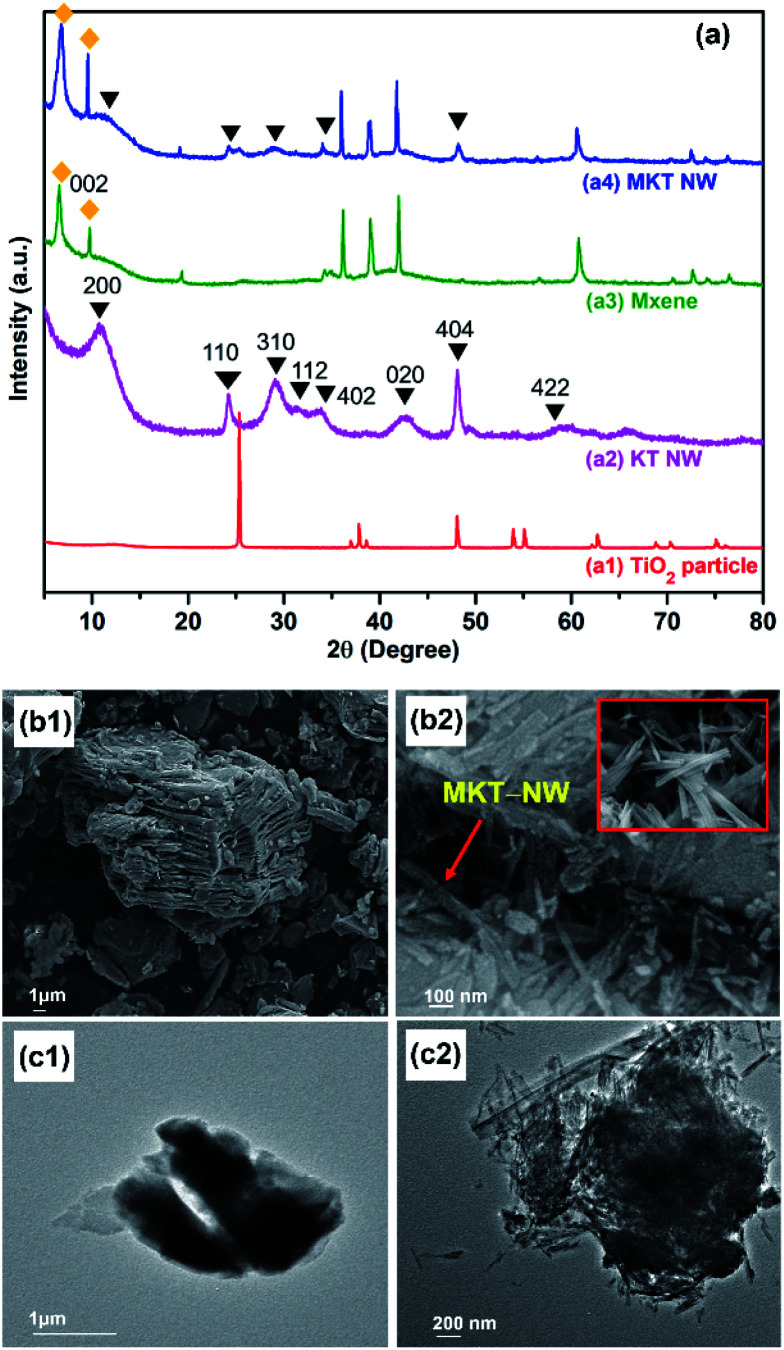
(a) WXRD patterns of (a1) TiO_2_ particle, (a2) KT-NW, (a3) MXene and (a4) MKT-NW composite, (b) FE-SEM images of (b1) MXene and (b2) MKT–KT NW (inset illustrates MKT-NW morphology), and (c) TEM images of (c1) MXene and (c2) MKT–KT NW composite.

In this work, KT-NW led to the formation of TiO_6_ octahedral by a zigzag pattern by connecting the edges and the potassium ion (K^+^) are trapped into this structure. In the KT NW synthesis process, longer Ti–O bonds might be attacked by –OH ions of strong aqueous KOH solution and were broken down but shorter Ti–O bonds remained unchanged, leading to the formation of Ti–O–K bonds.^21^ Thus, KT-NW was successfully formed by a simple hydrothermal method at 200 °C. From XRD patterns in Fig. 2(a), the pristine TiO_2_ nanoparticles showed intense peaks at 2*θ* of 25.37°, 37.87°, 48.16°, 54.00°, 55.21°, and 62.84° which are perfectly aligned to TiO_2_ anatase (COD ID 7206075) phase. The synthesized KT-NW corresponded to the crystal phase of K_2_Ti_6_O_13_ (AMCSD no: 0010602)^22^ and the intense peaks can be indexed as 200, 110, 310, 112, 402, 404, 020, and 422.

The starting material Ti_3_AlC_2_ (MAX) shows the standard XRD pattern (attached in Fig. S2†) as reported in the literature.^23^ MXene or Ti_3_C_2_T_*x*_ was obtained after chemically etching the MAX phase with HCl and LiF at 50 °C for 48 h, where HF was produced *in situ* in the reaction. The characteristic peaks for the MAX phase were disappeared but several peaks remained even after selective chemical etching of Al by HCl/LiF as the mild etching agent.^24^ The intense peak at 002 (9.5°) of MAX phase has reduced in MXene while a new characteristic sharp peak appeared at a low angle (6.4°) confirming the delaminated layered MXene structure. The XRD pattern of the MXene is in accordance with the literatures.^24,25^

The hydrothermal treatment at 120 °C led the formation of MKT-NW composite. The XRD pattern of MKT-NW composite ascribes the relevant peaks for MXene as well as KT-NW which confirmed the existence of the KT-NW in the MKT-NW composite. Furthermore, KT-NW would form less agglomeration compared to the TiO_2_ particle when introduced directly to MXene.^26^

FE-SEM images illustrates the formation of a layer structure of MXene that has been formed due to the delamination and exfoliation by *in situ* HF. Furthermore, EDS mapping has been conducted to determine the amount of chemically etched Al from the MAX phase during its transition to form MXene (Fig. S3†). TiO_2_ nanoparticles were completely transformed by a hydrothermal technique in the presence of concentrated KOH aqueous solution in the reaction system. Additionally, FE-SEM and TEM images shows the nanowire morphology of KT-NW on the surfaces of MXene as illustrates in Fig. 2(c2). The KT-NW has been compactly deposited on the surface of MXene as pleated nano wires by covering most of the surface area by developing a strong electrostatic interaction between them. The formation of MKT-NW had similar morphology with that of the work carried out to prepare MXene with TiO_2_ nanofibers.^27^

**Fig. 3:**
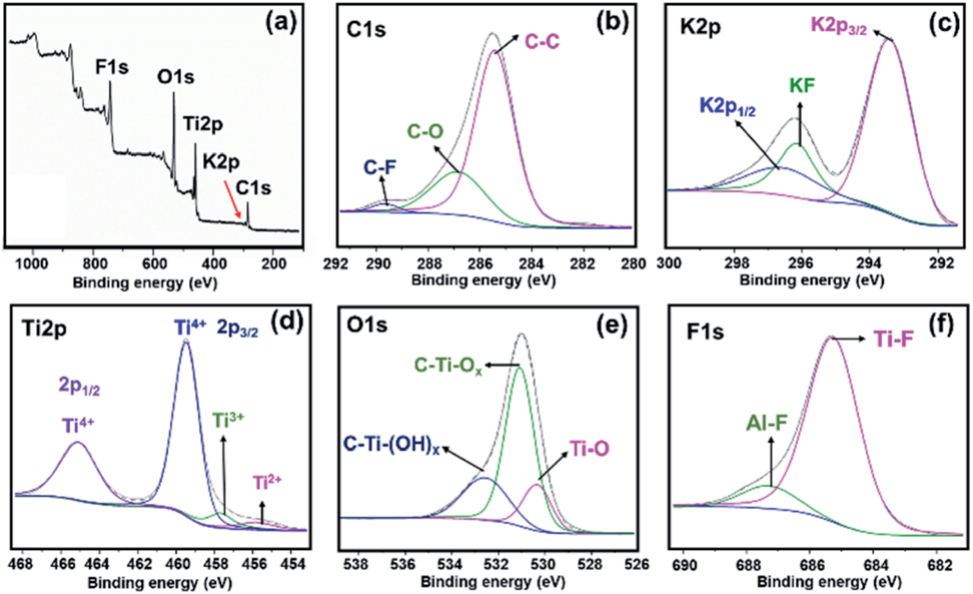
MKT-NW powder (a) XPS wide scan and deconvolution of (b) C 1s, (c) K 2p, (d) Ti 2p, (e) O 1s, and (f) F 1s regions.

In Fig. 3, the survey spectrum of MKT-NW shows the presence of C 1s, K 2p, Ti 2p, O 1s, and F 1s. All peaks precisely coincide with the binding energies given in the XPS database.^28,29^ The core level high resolution XPS analyses of MKT-NW powder shows the peaks of carbon (C 1s) is mainly found at 285.5, 286.8, and 289.7 eV which are assigned to sp^3^ C (C–C), C–O, and C–F, respectively. The K 2p signals (K 2p_3/2_ at 293.5 eV and K 2p_1/2_ at 296.8 eV) and KF at 296.1 eV were revealed to formation of potassium titanates from hydrothermal of TiO_2_ in alkaline media. The Ti 2p region shows two signals at Ti 2p_3/2_ (ranging from 453 to 462 eV) and Ti 2p_1/2_ (462 to 468 eV) with oxidation states (Ti^2+^, Ti^3+^ and Ti^4+^) according to the formation of mixed oxides (TiO_*x*_F_*y*_) and titanium oxycarbide (TiC_*x*_O_*y*_) which revealed non-uniform surface of MKT-NW. Deconvolution of O 1s also confirms the formation of Ti(iv) oxide and the presence of OH/O_*x*_ groups. The F 1s spectrum exhibited a strong signal at 685.2 eV which assigned to fluorine binding to the Ti carbide (Ti–F), thereby creating a C–Ti–F_*x*_ bond. The F 1s signal at the slightly higher binding energy of 687.2 eV can be attributed to C–Ti–OF_*x*_ or a fluorine bridging atom (C–Ti–F–Ti–C) or even an Al–F in MAX phase.^30^ This result is consistent with EDS mapping (Fig. S3†), about 2.9% wt of Al (or 2.6% atom) left in MXene after chemical etching from the MAX phase.

**Fig. 4:**
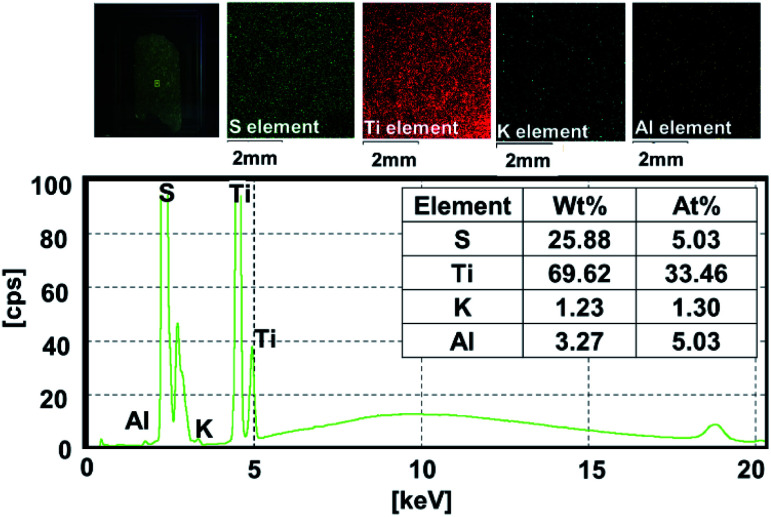
XRF elemental distribution of 12%MKT-NW/C-SPEEK hybrid composite membrane.

The surface morphology of pristine C-SPEEK and MKT-NW/C-SPEEK composites film revealed smooth and rough surface characteristics, respectively (Fig. S4†). The XRF elemental mapping of 12%MKT-NW/C-SPEEK hybrid composite PEM (Fig. 4) exhibited mainly S, Ti, K and Al with a uniform spatial dispersion within the polymer matrix. The distribution of Ti element within the polymer matrix was observed which were derived from MXene and KT-NW, along with the trace amount of K and Al elements.

### Membranes properties

The water molecule plays a vital role in the proton transfer mechanism as it can be used as a proton carrier to transfer protons from anode to cathode. The prepared SPEEK had high DS, thus the SPEEK membrane showed high water uptake and a swelling area which was later decreased after cross-linking with EG.^20^ But, it was observed that there was an increase in water uptake after introducing MKT-NW into the C-SPEEK membrane; which was due to the hydrophilic nature of MXene and KT-NW.^15^ However, the swelling area was decreased with the increase of MKT-NW which may be due to the physical interaction of –SO_3_H of SPEEK and –OH of EG with MXene and KT-NW. The water uptake of 20% MKT NW/C-SPEEK was low and showed a high swelling area, which may be due to the accumulation of the MKT-NW.^31^ The water uptake and swelling area of all the prepared membranes are given in Table 1.

**Table 1:** Water uptake, swelling area, and proton conductivity of hybrid composite membranes

Samples	Water uptake (%)	Swelling area (%)	Proton conductivity (S cm^−1^)
SPEEK	23.40	21.01	0.0076
C-SPEEK	10.00	18.91	0.0034
5%MKT-NW/C-SPEEK	21.05	16.74	0.0038
10%MKT-NW/C-SPEEK	47.36	10.35	0.0061
12%MKT-NW/C-SPEEK	48.57	8.15	0.0097
14%MKT-NW/C-SPEEK	51.42	12.46	0.0078
20%MKT-NW/C-SPEEK	38.88	16.73	0.0066

**Fig. 5:**
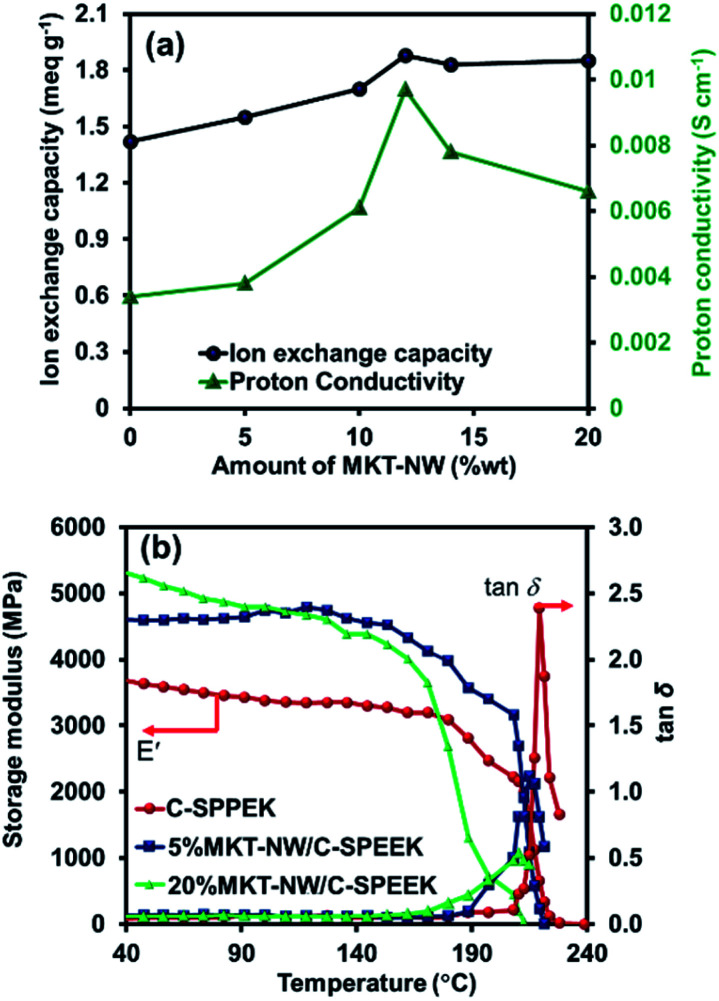
(a) Ion exchange capacity and proton conductivity of MKT-NW/C-SPEEK composite membranes with different amount of MKT-NW and (b) storage modulus (*E*′) and tan *δ* of membranes.

In the proton exchange membrane, ion exchange capacity is one of the important factors that insight the ionic sites of each PEM which relates to the proton conductivity. Here, C-SPEEK membrane had low IEC of 1.42 meq g^−1^ due to the cross-linking of ethylene glycol with the –SO_3_H group of SPEEK which caused the reduction of acidic sites.^31^ An IEC increased after MKT-NW was incorporated with C-SPEEK and further increased with an increasing in the amount of MKT-NW^32^ The 12%MKT-NW/C-SPEEK composite hybrid membrane performed highest IEC of 1.88 meq g^−1^ according to the well distribution of MKT-NW within the membrane compared to the others. Moreover, a further decrease in the IEC was observed at the amount of MKT-NW more than 12% which may be due to the agglomeration of the inorganic filler.^33^ The IEC of all the prepared membrane is presented in Fig. 5(a).

The proton conductivity of all MKT-NW/C-SPEEK composite membranes at room temperature is shown in Table 1. The pristine SPEEK membrane exhibits high proton conductivity according to the presence of a large number of –SO_3_H group in the hydrophilic domain part of the polymer. In addition, the casted membrane using ethanol and water (SPEEK) which have lower interaction as compared to the other casted SPEEK membranes using an organic solvent, leading to high proton conductivity of membranes.^33^ However, the conductivity thereby decreased as it was cross-linked with EG because the number of –SO_3_H group would become lower as it will be cross-linked with –OH group of EG. Furthermore, after MKT-NW was incorporated with C-SPEEK, the proton conductivity increased as shown in Fig. 5(a). It was due to the hydrophilic nature of MXene as well as KT-NW which absorbed more water molecules that acts as the proton carrier in vehicular type mechanism. Nevertheless, the presence of –SO_3_H from SPEEK and –OH from composite as well as EG would develop an extensive interconnected hydrogen bond network and shorten the distance for faster proton transfer pathway, through Grotthuss mechanism, thereby increasing proton conductivity.^35,36^ The proton conductivity increased with an increasing in the amount of MKT-NW and the addition of MKT-NW more than 12% decreased the proton conductivity, which may be due agglomeration and non-uniform distribution of the composite. The 12%MKT-NW/C-SPEEK exhibits proton conductivity of 0.0097 S cm^−1^ which was higher than that of the commercially available Nafion-212 (0.0093 S cm^−1^) due to the well distribution of composite in the polymer matrix.

### Dynamic mechanical strength

To elucidate the effect of MKT-NW composite on C-SPEEK dynamic mechanical analysis, the composite membrane with 5%MKT-NW and 20%MKT-NW were considered as the minimum and maximum MKT-NW content, respectively. The storage modulus of C-SPEEK was increased when 5% MKT-NW was added whilst 20% MKT NW led to the further increment of storage modulus as shown in Fig. 5(b) and Table 2. The highest storage modulus of 20%MKT-NW/C-SPEEK was 5436 MPa which was 1.44 times higher than that of C-SPEEK (3762 MPa). Thus, the addition of MKT-NW improved the storage modulus of C-SPEEK, which was due to the strong interaction of highly hydrophilic MKT-NW developed with C-SPEEK. Hence, rendering improved mechanical strength to the MKT-NW/C-SPEEK hybrid composite PEM.

**Table 2:** Storage modulus (*E*′) of the membranes at different temperature

Samples	Storage modulus (*E*′) (×10^3^ MPa)					*T*_g_ (°C)
	30 °C	50 °C	100 °C	150 °C	200 °C	
C-SPEEK	3.76	3.62	3.38	3.29	2.36	221
5%MKT-NW/C-SPEEK	4.37	4.59	4.67	4.60	3.85	222
20%MKT-NW/C-SPEEK	5.43	5.25	4.82	4.60	3.86	223

The glass transition temperature (*T*_g_) of C-SPEEK, 5%MKT-NW/C-SPEEK, and 20%MKT-NW/C-SPEEK membranes were depicted using the plot of tan *δ* various with temperature. The *T*_g_ of C-SPEEK membranes was 221 °C while the addition of MKT-NW leads to the small increment of *T*_g_ membrane (∼1 °C for 5%MKT-NW and ∼2 °C for 20%MKT-NW) because of the formation of dense intermolecular hydrogen bonding within MKT-NW and C-SPEEK.

C-SPEEK had higher *T*_g_ value than SPEEK membranes as reported by X. Zhang *et al.* (181 °C)^37^ after cross-linking with EG and the addition of MKT-NW does not bring noteworthy change in the *T*_g_ of C-SPEEK membranes. Although it is worth mentioning that MKT-NW had significantly affected to the mechanical strength of C-SPEEK membranes as observed from the storage modulus data at the different temperatures as summarized in Table 2. The C-SPEEK using EG as a cross linker had shown higher storage modulus as compared to the cross linked membrane using polyethylene glycol (PEG) or 1,4-cyclohexane dimethanol (CDM) as a cross linker.^38^ Additionally, the MKT-NW/C-SPEEK had shown higher storage modulus than SPEEK composite membranes which was due to the cross-linking of SPEEK with EG as well as MKT-NW composite to form a strongly hydrogen-bonded within the hybrid composite membranes.^39^

### Photocatalytic activity of hydrogen production from water

**Fig. 6:**
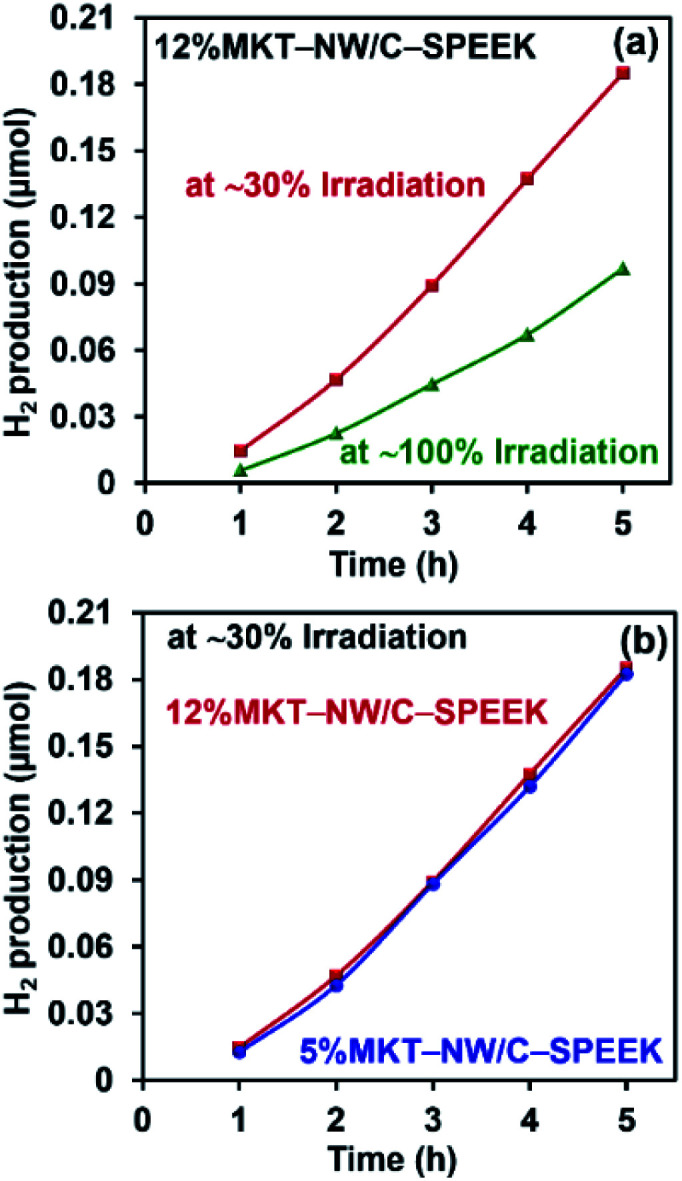
(a) H_2_ production of 12%MKT-NW/C-SPEEK with ∼30% irradiation and ∼100% irradiation of the membrane area by UV light and (b) effect of MKT-NW content to the photocatalytic activity at ∼30% irradiation.

The prepared MKT-NW/C-SPEEK hybrid composite membrane showed photocatalytic activity due to the presence of MKT-NW in the polymer matrix. The optimized composite membrane (12%MKT-NW/C-SPEEK) was used to determine the effect of UV light intensity on photocatalytic H_2_ production. Initially, the UV light was located close to the membrane in which ∼30% of the total surface of the membrane was irradiated. And the light was moved further from the membrane to irradiate the total area of the membrane (∼100%). The intensity of the light depends upon the distance of the light source and the exposed area of the membrane. The MKT-NW got more excited when the UV light intensity was higher (irradiated ∼30% of the area), and this was compared with the membrane which had lower intensity of UV light (irradiated ∼100% of the membrane). A significant effect on the photocatalytic H_2_ production from water using the membrane had been noticed as the ∼30% irradiated membrane produced 0.185066 μmol of H_2_ within 5 h and ∼100% irradiated membrane produced 0.096649 μmol H_2_ within 5 h as shown in Fig. 6(a).

As the ∼30% irradiated membrane with higher intensity could produce more H_2_ gas, this irradiation was considered to study the effect of the MKT-NW composite in the photocatalytic activity of membranes. Two-hybrid composite membranes namely – 12%MKT-NW/C-SPEEK and 5%MKT-NW/C-SPEEK had been selected to study the photocatalytic H_2_ production. In order to know the effect of MKT-NW content to the photocatalytic activity, 5% and 12%MKT-NW/C-SPEEK were used in this study. It had been observed that both membranes could produce H_2_ linearly with respect to time within 5 h. The H_2_ production of 12%MKT-NW/C-SPEEK was slightly different from 5%MKT-NW/C-SPEEK. This might depend on the higher amount of MKT-NW in 12%MKT-NW/C-SPEEK composite PEM as shown in Fig. 6(b).

**Scheme 3:**
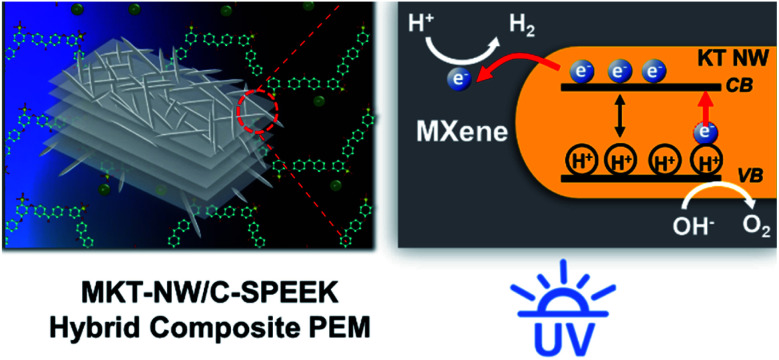
Illustration of photolytic production of H_2_ from water using MKT-NW/C-SPEEK membrane under UV light.

When UV light was irradiated the KT-NW in the polymer matrix, KT-NW would be excited to produced electron–hole pair (e^−^ and H^+^). Meanwhile, the valance band (VB) of KT-NW would remain with H^+^ since the e^−^ in the conduction band (CB) of KT-NW would swiftly move towards MXene and accumulated on the surface because of the superior metallic and electronic properties. Then, a Schottky barrier would develop on the interface of MKT-NW composite blocking the back transfer of e^−^ to KT-NW. And the H^+^ in the aqueous methanol solution would undergo reduction with the help of the photo-induced e^−^ on MXene producing H_2_ (ref. 13–40) as presented in Scheme 3. Thus, making this hybrid composite PEM as a photocatalytic active membrane that produces H_2_ from water without the addition of any noble metals as co-catalysts in the same way as that of the photocatalytic membranes prepared by immobilizing rGO/TiO_2_ into Nafion membranes.^41^

**Fig. 7:**
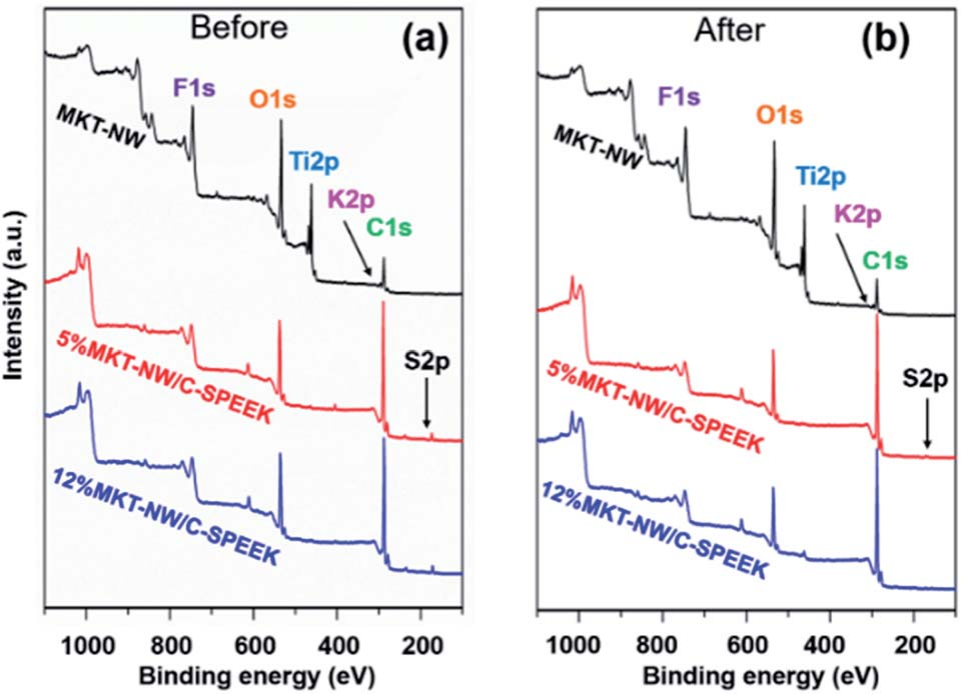
Wide-scan X-ray photoelectron spectroscopy (XPS) spectra (a) before H_2_ evolution testing and (b) after H_2_ evolution testing. F: fluorine; O: oxygen; Ti: titanium; K: potassium; C: carbon; S: sulfur.

The membranes before and after H_2_ evolution testing under UV irradiation were characterized by XPS wide scan and core level high resolution. Fig. 7 illustrates XPS spectra for MKT-NW and their composites with SPEEK. The spectra of MKT-NW (before and after H_2_ evolution testing) shows the signals of F 1s, O 1s, Ti 2p, K 2p, and C 1s. For the MKT-NW/SPEEK composites, new peak of S 2p region ranging from 164 to 176 eV, which revealed the presence of sulfonyl entirely in the composite membranes, and the disappearance of the peak due to F 1s were observed. The depth of the XPS surface analysis is about 10 nm, which is the limitation of XPS characterization. The deconvolution of F 1s, O 1s, Ti 2p, K 2p, C 1s, and S 2P are presented in Fig. S5 and S6† and summarized in Table 3. XPS peak due to S decreased after the H_2_ evolution testing, suggesting the degradation *via* loss of sulfonic bonds within the SPEEK membrane and further structural deformation after long term exposure to UV irradiation.

**Table 3:** The apparent atomic compositions of the initial MKT-NW and the composite samples as determined by XPS^a^

Materials	Atomic composition (%)					
	C 1s	O 1s	Ti 2P	F 1s	K 2p	S 2p
MKT-NW	36.2	44.7	15.6	1.35	2.16	0.00

**5%MKT-NW/C-SPEEK**						
Before testing	77.4	19.4	0.28	N/A	0.40	2.58
After testing	71.5	27.1	0.15	N/A	0.66	0.63

**12%MKT-NW/C-SPEEK**						
Before testing	78.8	17.7	0.24	N/A	0.72	2.51
After testing	88.8	9.85	1.03	N/A	0.15	0.22

a N/A is not applicable according to the depth of the XPS surface analysis is 10 nm. H_2_ evolution testing for 5 h under UV irradiation.

To predict the photophysical and photochemical properties, absorption and diffuse reflectance spectra of all composites were investigated by UV-Vis spectrometer (UV-2600, Shimadzu) at the range of 200 to 800 nm from the transmission and diffuse-reflectance modes as shown in Fig. S7.† Apparent differences in the spectra shapes were seen depending on the composition, confirming the successful incorporation of potassium titanate and MXene. However, it is difficult to isolate the absorption to be ascribed into the components (polymer and additives) due to the overlapping. More systematic studies will be done in the future studies.

Table 4 presents composites activity over the reported SPEEK-functionalized materials.^11,20,42–44^ It was found that the MKT-NW/C-SPEEK composite membrane of this work had shown quite a good proton conductivity at room temperature while the other SPEEK composite membranes were studied at higher temperature. Our recent work assumed that the present composite membrane was able to transfer proton faster without being influenced by temperature because of the hydrophilic nature of MKT-NW as well as the hydrogen bonding. Additionally, the MKT-NW/C-SPEEK possessed photocatalytic activity and able to produce H_2_ from the membrane.

**Table 4:** Comparison of present composites activity over the reported SPEEK-functionalized materials

Membranes	Proton conductivity (S cm^−1^)	Temp. (°C)	Ref.
7.5%TiO_2_–SO_3_H/SPEEK	0.0138	30	42
10%Ti_3_C_2_T_*x*_/SPEEK	0.1470	40	11
4%CNCs/C-SPEEK	0.1860	95	20
5%Clay/C-SPEEK	0.0197	80	43
8%SSi-GO/SPEEK	0.1626	65	44
12%MKT-NW/C-SPEEK	0.0097	25	This work

## Conclusions

MKT-NW/C-SPEEK hybrid composite PEM had been successfully prepared. MKT-NW was prepared by a hydrothermal method and was used as the filler to improve the physicochemical properties of membranes. The MKT-NW led to improve the mechanical strength of the C-SPEEK membrane. The 12%MKT-NW/C-SPEEK had the highest proton conductivity of 0.0097 S cm^−1^ with the IEC of 1.88 meq g^−1^. Besides, it was a photocatalytic membrane which was able to produce 0.1850 μmol of H_2_ in 5 h by photocatalysis of water. Consequently, the prepared MKT-NW/C-SPEEK hybrid composite PEM is a smart PEM with dual properties showing the photocatalytic activity of H_2_ production from water, along with the proton conductivity improvement. The overall process utilized was green and sustainable as the mixture of ethanol and water was used as the solvent for PEM casting rather than using hazardous organic solvents. Thus, MKT-NW/C-SPEEK hybrid composite PEM could be utilized in many applications namely; PEM for PEM fuel cells, direct methanol fuel cells, microbial fuel cells and PEM water electrolysis or artificial photosynthesis, which may induce a useful impact on the field of clean and green energy.

## Abbreviations

MXene (Ti_2_C_2_T_*x*_): Titanium carbide
KT-NW (K_2_Ti_6_O_13_): Potassium titanate nanowire
SPEEK: Sulfonated polyether ether ketone
EG: Ethylene glycol
C-SPEEK: Cross-linked sulfonated polyether-ether ketone
MKT-NW/C-SPEEK: MXene-potassium titanate nanowire/cross-linked sulfonated polyether-ether ketone
DS: Degree of sulfonation
PEM: Proton exchange membrane
IEC: Ion exchange capacity
HER: Hydrogen evolution reaction

## Conflicts of interest

There are no conflicts to declare.

## Supplementary Material

RA-011-D0RA09935J-s001
