# Supplementary material for: MXene potassium titanate nanowire/sulfonated polyether ether ketone (SPEEK) hybrid composite proton exchange membrane for photocatalytic water splitting
Source: RSC Adv. 2021 Mar 1;11(16):9327–35. doi: 10.1039/d0ra09935j (PMC8695234; doi:10.1039/d0ra09935j)
Supplement: RA-011-D0RA09935J-s001 [file RA-011-D0RA09935J-s001.pdf]

Electronic Supplementary Material (ESI) for RSC Advances.
This journal is © The Royal Society of Chemistry 2021

Electronic Supplementary Information(ESI)

MXene Potassium Titanate Nanowire / Sulfonated Polyether Ether Ketone (SPEEK) Hybrid Composite Proton Exchange Membrane for Photocatalytic Water Splitting

Preeti Waribam[a], Kanticha Jaiyen[a], Chanatip Samart[a,b], Makoto Ogawa[c], Guoqing Guan[d], Suwadee Kongparakul[a,b]*

[a]*Department of Chemistry, Faculty of Science and Technology, Thammasat University, Pathumthani 12120, Thailand*

[b]*Bioenergy and Biochemical Refinery Technology Program, Faculty of Science and Technology, Thammasat University 12120, Thailand*

[c]*Department of Chemical and Biomolecular Engineering, School of Energy Science and Engineering, Vidyasirimedhi Institute of Science and Technology, Rayong 21210, Thailand*

[d]*Institute of Regional Innovation, Hirosaki University, Aomori 030-0813, Japan*

Corresponding author

Email address: ksuwadee@tu.ac.th

Table of Contents **Page**

1. Characterization of materials and membranes S-3
2. **Fig.S1** ATR- FTIR spectra of PEEK and SPEEK S-4
3. **Fig. S2** WXRD patterns of MAX phase and MXene S-4
4. **Fig.S3** EDS mapping of (a) MAX phase and (b) MXene S-5
5. **Fig.S4** FE- SEM images of surface morphology of (a) C- SPEEK and (b) 12%MKT-NW/C- SPEEK hybrid composite PEM S-5
6. **Fig.S5** Deconvolution of 5%MKT-NW/C- SPEEK hybrid composite PEM before and after H_2 evolution testing for 5h S-6
7. **Fig.S6** Deconvolution of 12%MKT-NW/C- SPEEK hybrid composite PEM before and after H_2 evolution testing for 5h S-6
8. **Fig.S7** Absorption spectra and diffuse reflectance spectra of (a) C-SPEEK MKT- NW/C-SPEEK composite films contain 5 – 20%wt of MKT-NW (b) – (f) S-7
9. **Table S1** Photocatalytic H_2 production of MKT 12%/C-SPEEK hybrid composite PEM under different irradiation of UV light (~30% and ~100% of membrane irradiation) S-7
10. **Table S2** Photocatalytic H_2 production of 12%MKT-NW/ C-SPEEK and 5%MKT-NW/ C-SPEEK hybrid composite PEM (~30% of membranes irradiated) S-8

Characterization of materials and membranes

Proton nuclear magnetic resonance spectroscopy (^1H NMR) was used to determine the degree of sulfonation of SPEEK. SPEEK particle was dissolved using deuterated dimethyl sulfoxide (DMSO)-d_6) and it was recorded using Bruker Avance III HD 600 MHz NMR spectrometer. All chemical shifts were described in parts per million (ppm) with reference to tetramethylsilane (TMS) at 0.00 ppm. And the equation used in the calculation as reported in reference[4] is given below-

$$\frac{X}{(12-2X)} = \frac{AH_E}{\sum AH_{A,A',B,B',C,D}} \quad (1)$$

Where, X = no. of H_E protons per repeat units

AH_E = peaks area under H_E proton and

$\sum AH_{A,\ A',\ B,\ B',\ C,\ D}$ = integrated peak areas of other aromatic protons

Water uptake and swelling area of the membrane were determined as described below. A dry circular-shaped membrane (1 cm diameter) was weight (W_{dry}) and area (A_{dry}) was measured. Then, immersed in 20 ml DI, which was kept at room temperature for 24 h. After 24 h, it was taken out and immediately wiped off the water on the surface and wet weight (W_{wet}) and weight area (A_{wet}) were recorded. The water uptake and swelling area were calculated using the formula given below-

$$\text{Water uptake (\%)} = \frac{(W_{wet}-W_{dry})}{W_{dry}} \times 100\% \quad (2)$$

$$\text{Swelling area (\%)} = \frac{(A_{wet}-A_{dry})}{A_{dry}} \times 100\% \quad (3)$$

The ion exchange capacity (IEC) value of the membrane was determined using acid-base titration. The dry and pre weighted sample (Wm) were immersed in 2.0 M NaCl solution at room temperature for 24 h to completely liberate H^+ by exchanging with Na^+. Then, the liberated H^+ was then titrated against 0.01 M NaOH solution using phenolphthalein as an indicator.

The IEC was calculated by the equation-

$$\text{IEC (meq g}^{-1}) = \frac{V_{NaOH} \times C_{NaOH}}{W_m} \quad (4)$$

where V_{NaOH},C_{NaOH}, and W_m are the volumes of NaOH solution (mL) in the titration, the concentration of NaOH, and the weight of the dry membrane (g), respectively. The IEC value is the average value of the three samples.

Proton conductivity of membranes was determined at room temperature using electrochemical impedance spectroscopy (EIS) (Corrtest CS310). In this method, membranes were immersed in DI for 24 h to make it completely hydrated. And conductivity of proton was calculated using the formula-

$$\sigma = \frac{l}{A \times R} \quad (5)$$

Where, σ = The proton conductivity (Scm^{-1}), *l* = Thickness of the membrane (cm), A = Area of the membrane (cm^2) and R = Resistance of the membrane (Ω).

Absorption and diffuse reflectance spectra of all membranes were analyzed using UV-Vis spectrometer(UV-2600, Shimadzu) at the range of 200 to 800 nm.

FTIR of PEEK and SPEEK

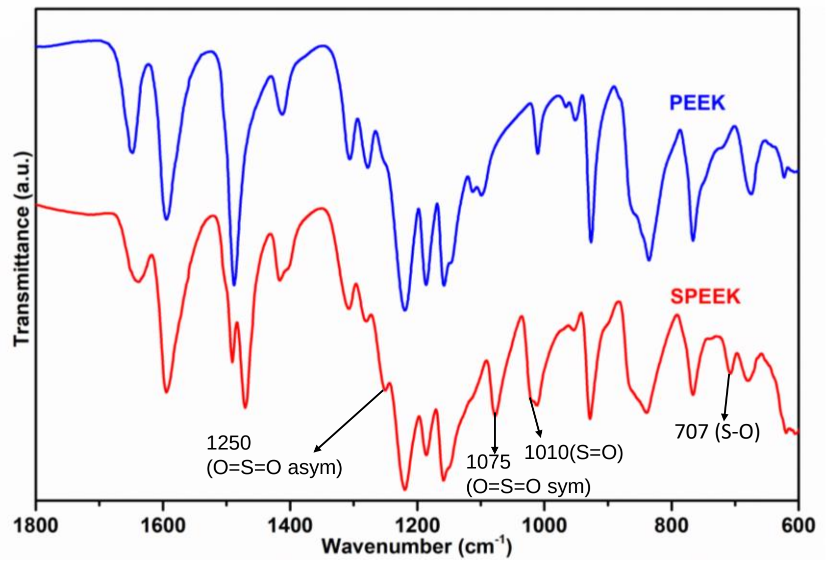

Fig.S1 ATR- FTIR spectra of PEEK and SPEEK.

WXRD of MAX and MXene

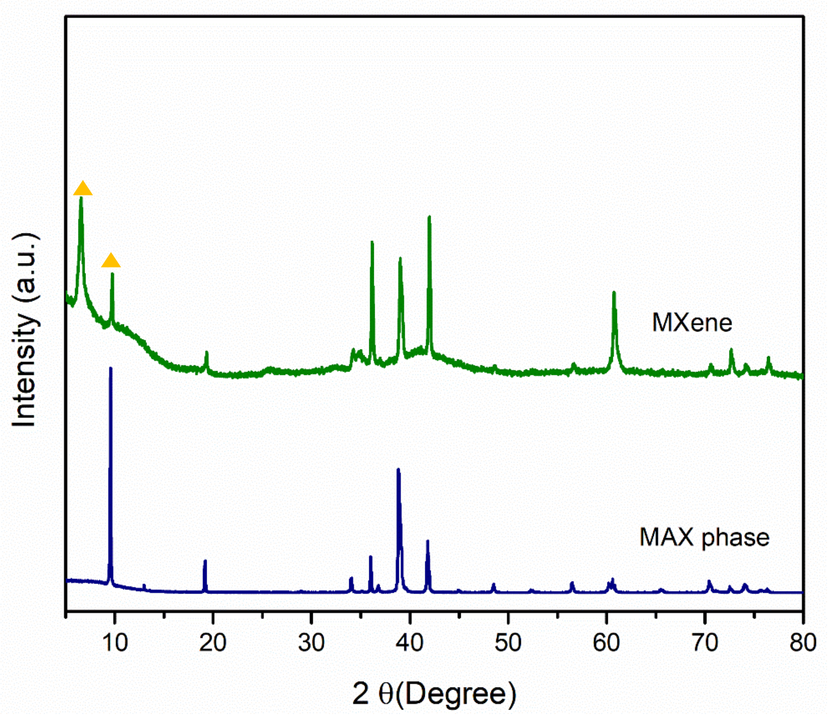

Fig. S2 WXRD patterns of MAX phase and MXene.

EDS mapping of MAX phase and MXene

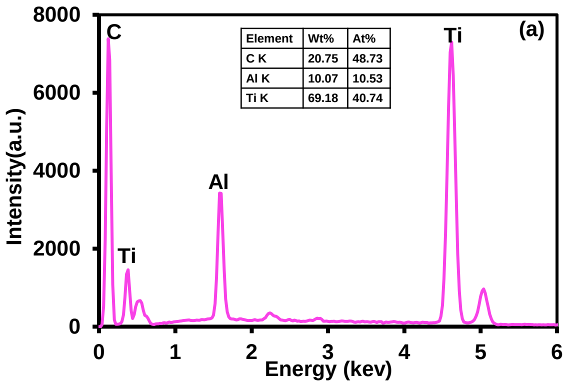

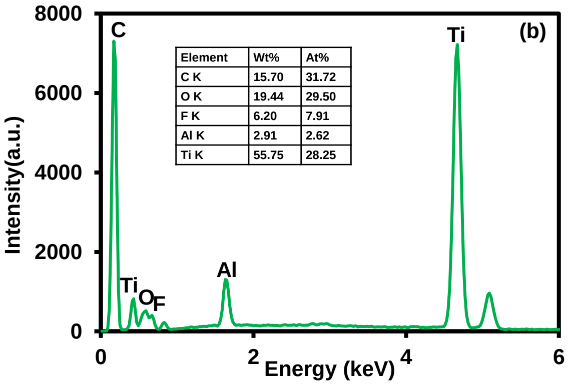

Fig.S3 EDS mapping of (a) MAX phase and (b) MXene.

FE- SEM images of surface morphology of C- SPEEK and 12%MKT-NW/C- SPEEK

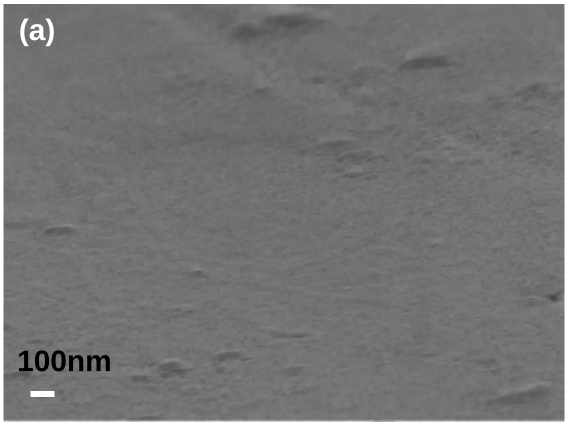

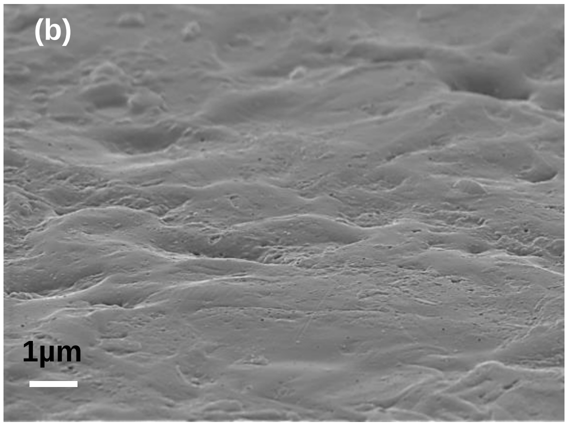

Fig.S4 FE- SEM images of surface morphology of (a) C- SPEEK and (b) 12%MKT-NW/C- SPEEK hybrid composite PEM.

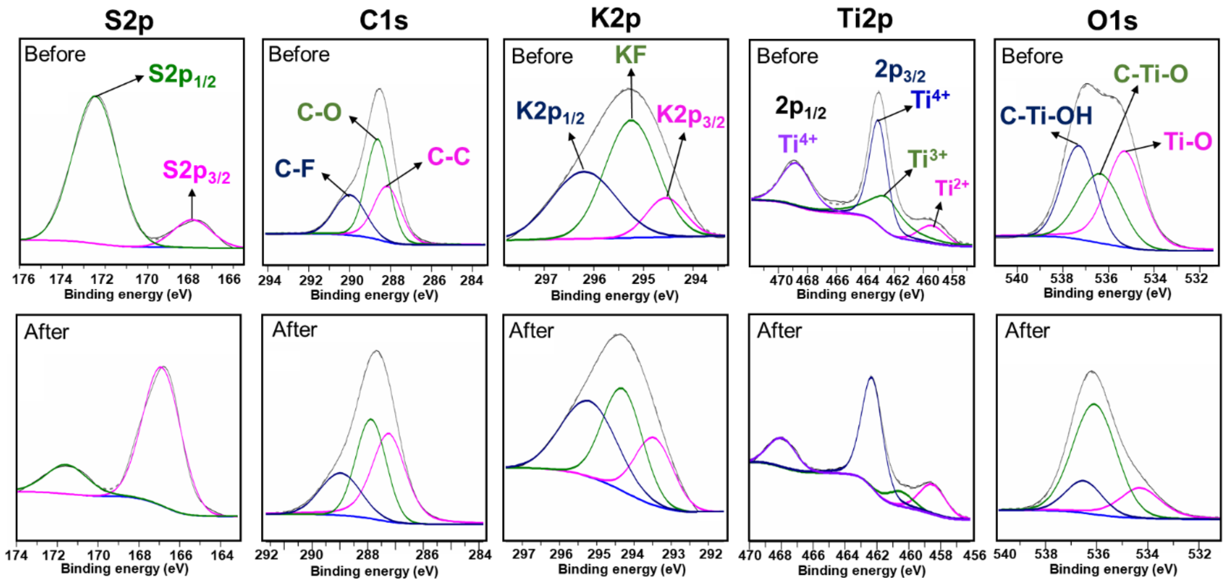

Fig.S5 Deconvolution of 5%MKT-NW/C- SPEEK hybrid composite PEM before and after the H_2 evolution testing for 5h.

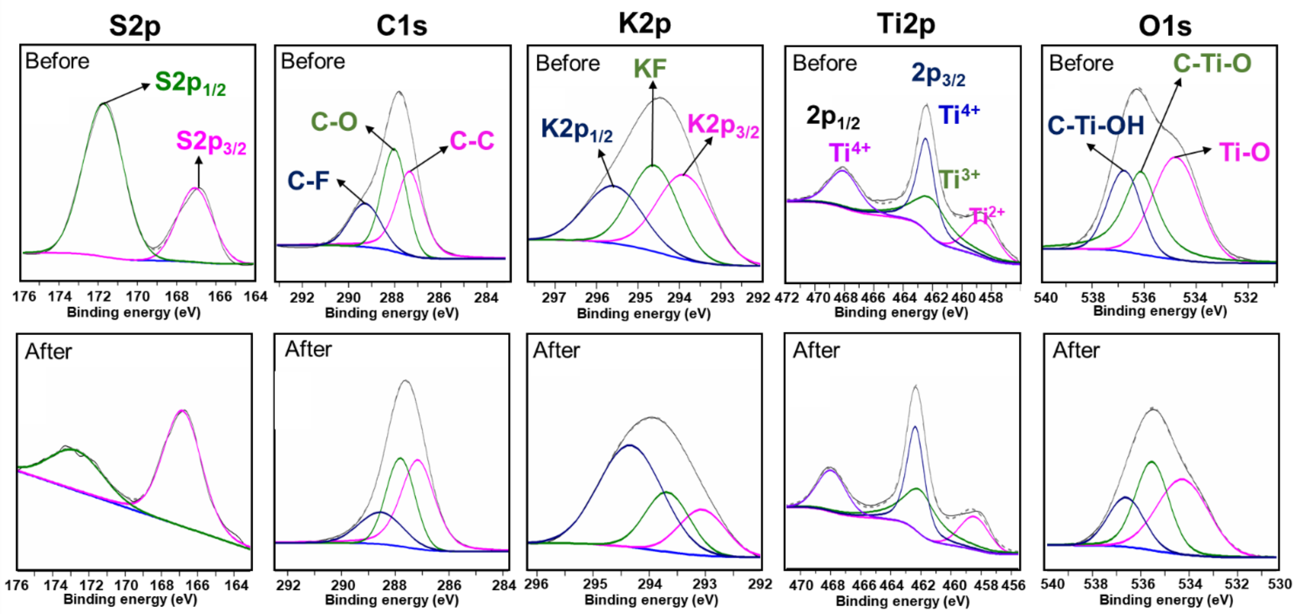

Fig.S6 Deconvolution of 12%MKT-NW/C- SPEEK hybrid composite PEM before and after the H_2 evolution testing for 5h.

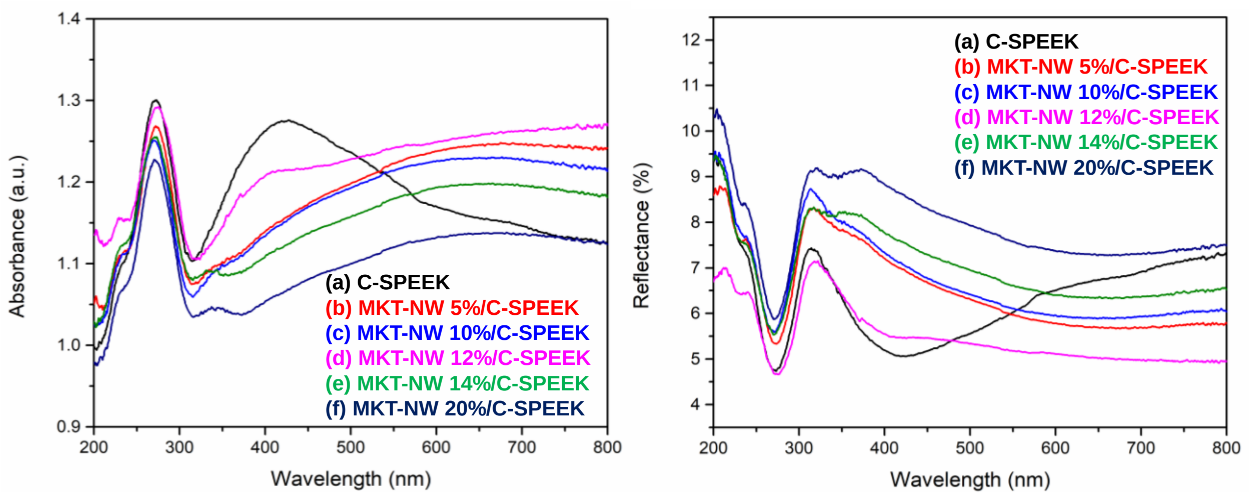

Fig.S7 Absorption spectra and diffuse reflectance spectra of (a) C-SPEEK, and MKT- NW/C-SPEEK composite films contain 5 – 20%wt of MKT-NW (b) – (f).

Table S1 Photocatalytic H_2 production of 12%MKT-NW/C-SPEEK hybrid composite PEM under different irradiation of UV light (~30% and ~100% of membrane irradiation)

Sl. No	Time (h)	MKT NW 12%/ C-SPEEK H_2 production (~30% irradiation) (µmol)	MKT NW 12%/ C-SPEEK NW H_2 production (~100% irradiation) (µmol)
a.	1	0.014658	0.005635
b.	2	0.046844	0.022372
c.	3	0.089236	0.044492
d.	4	0.137354	0.066976
e.	5	0.185066	0.096649

Table S2 Photocatalytic H_2 production of 12%MKT-NW/ C-SPEEK/ and 5%MKT-NW/ C-SPEEK/hybrid composite PEM (~30% of membranes irradiated)

Sl. No	Time (h)	MKT NW 12%/ C-SPEEK H_2 production (µmol)	MKT NW 5 %/C-SPEEK H_2 production (µmol)
a.	1	0.014658	0.012509
b.	2	0.046844	0.042616
c.	3	0.089236	0.088144
d.	4	0.137354	0.131915
e.	5	0.185066	0.182595
